# CT scanning and questions about benefit versus risk

**DOI:** 10.1002/acm2.70179

**Published:** 2025-07-15

**Authors:** Cynthia H. McCollough, Rebecca Millman

**Affiliations:** ^1^ Department of Radiology Mayo Clinic College of Medicine and Science Rochester Minnesota USA; ^2^ Department of Radiology University of Colorado School of Medicine Aurora Colorado USA

Computed tomography (CT) was invented over 50 years ago and is considered one of the greatest medical advances of the 20th century.^1^ Physicians depend on CT in myriad medical scenarios, from diagnosing and treating cancer patients to determining whether a surgery is necessary. CT increases diagnostic accuracy and decreases patient mortality.^2^, ^3^, ^4^, ^5^, ^6^, ^7^, ^8^

A recent publication^9^ estimated that up to 5% of all future cancers in the U.S. may be caused by CT scans. The paper, and the ensuing media coverage, reinforced perceptions that CT scans are risky medical procedures that should be avoided. This perception is due in large part to similar papers by some of the same authors^10^, ^11^, ^12^ and the alarmist reporting of these papers by large media outlets.

It is therefore essential that medical personnel, including medical physicists, be able to discuss this topic in a reassuring and well‐informed manner when patients question the safety of a prescribed CT (or other exam or procedure involving ionizing radiation). Toward that end, the AAPM produced a communication guideline entitled *Radiation and Medical Imaging: Communicating Clear Answers to Top Questions*.^13^ The guide was written to help health professionals explain the benefits and risks of medical imaging to policy makers, care providers, patients, family members, and the public. In this editorial, we provide additional information for answering questions regarding cancer risk from CT.

First, it must be noted that the methods used by Smith‐Bindman et al.^9^ are fundamentally mathematical in nature; they assume a causal relationship between CT and cancer rather than prove it, and they provide no direct evidence of any single person getting cancer from a CT scan. They estimate 103 000 additional cancers might occur per 93 000 000 CT exams (0.1%) compared to what would otherwise be expected based on organ doses from modern CT exams, numbers of CT scans performed, and the BEIR VII^14^ organ risk coefficients (scaled down from 100 mGy). Notably, the estimates are derived from risk coefficients published in BEIR VII—coefficients that were derived from very different populations, including populations with much higher doses than for CT.^14^

While BEIR VII is an important document, there are considerable limitations to the risk coefficients it provides. Data based on human exposures to radiation are extremely limited, making it necessary to form risk estimates from a combination of data from higher dose exposures (well above 100 mGy) and animal and cellular studies, most of which were performed with radiation exposures on the order of Gy rather than the 10s of mGy used in medical imaging exams. More recent risk estimates from medical exposures (specifically CT) suffer from multiple limiting or confounding factors, including a lack of patient‐specific dose estimates and medical records, increasing the uncertainty of any derived risk values.^15^ Additionally, risk estimates derived from medical populations may suffer from what is known as reverse causality—did the CT cause a cancer or did the symptoms for which the CT was performed indicate a future cancer.

Importantly, the estimates in Smith‐Bindman et al.^9^ assume that a person's lifetime risk of developing cancer after having a CT scan is the same for a healthy person—where the exam is not justified—as it is for a sick or injured person needing a CT exam. Brenner et al. showed that patients with significant existing disease have a far lower risk of developing cancer from a CT exam than a person with no disease,^16^ since a serious pre‐existing condition (prior to the CT) can cause death before cancer from a CT would have time to develop. Hence, the estimates in the Smith‐Bindman paper greatly overestimate radiation risk since the individuals in the study who received a CT scan already had symptoms or a diagnosis of some sort of injury or disease. Even Smith‐Bindman has previously reported that among patients who received “high” CT doses (total effective doses ≥ 100 mSv over 5 years), 80% were ordered because of suspected or known malignancy.^17^ Hence a great many of the CT exams included in the 2025 Smith‐Bindman et al. study likely occurred in patients who already had cancer. The BEIR VII risk estimates were for individuals with full life expectancies and cancer rates similar to the general population^18^ and do not apply to a patient population.

Mataac et al.^19^ showed that only 50% of patients who receive multiple CT scans (and hence “high” doses) are alive 2 years after their CT scans. For these patients, the cause of death must be due to their underlying disease or injury since radiation takes much longer than 2 years to cause a solid cancer, typically from 5 to 40 years, and at least 2 years to cause a leukemia. Thus, patients in the Smith‐Bindman cohort who received the highest doses had substantially decreased lifespans *before* having a CT scan and would likely die before any radiation induced injury could express itself.

Discussions of CT safety must consider an additional aspect of risk—the risk from medical decisions made without the information provided by a CT scan. CT scans guide medical care and improve health outcomes. The same article that reported rates of malignancy among patients receiving CT scans found that 7% of patients who received higher CT doses were imaged due to acute, critical conditions such as aortic dissection, aneurysm, or high energy trauma, where there is a significant risk of death if the condition is not accurately diagnosed and treated.^17^ While not immediately life‐threatening, other indications were for conditions that pose substantial health risks, such as pancreatitis, non‐acute vascular disease, and failed surgery (13%).^17^ Without the diagnostic information from a CT scan, these patients risk poorer management of their condition, leading to worse medical outcomes.

The American Cancer Society estimates that one in two men and one in three women in the United States will be diagnosed with cancer at some point in their lives.^20^ In comparison to these background cancer rates, an increase in risk from a CT (estimated to be ∼0.1% for a typical body exam and lower for head exams) is small compared to the large benefits that have been shown time and again from CT:

1. In a study of almost 12 000 patients who received surgery to remove their appendix, about 10% of patients imaged using ultrasound received unnecessary surgery due to an incorrect diagnosis. With CT, the number of unnecessary surgeries fell to 2.5%.^2^, ^3^
2. Use of CT in the emergency department changed the diagnosis and patient management in 20%–50% of evaluated patients.^4^
3. In a study of patients with abdominal pain, weight loss, or blood in their urine, CT changed the doctor's initial diagnosis in 50% of patients and changed clinical management in 35%–54% of patients.^5^
4. A 20% decrease in lung cancer deaths was observed in smokers and ex‐smokers who received low‐dose CT scans of their lungs compared to those who had a chest x‐ray.^6^
5. Following conservative guidelines for use of childhood head CT, serious positive findings occurred in 4%–5% of imaged patients. Hence, many more patients benefit from head CT (400‐500 per 10 000) than the 1 per 10 000 brain cancers *potentially* caused by radiation from the head CT.^7^, ^21^
6. In a study of 92 800 patients with blunt traumatic injury, CT reduced negative laparotomy rates by 11% and decreased mortality from 22.6% to 14.1%.^8^

In summary, the risk from CT is small and unproven, while the benefits are unequivocal. If patients considering having a CT ask about potential radiation risks, the large benefits of undergoing a medically justified CT exam must be included in the discussion. Hopefully the information provided here can assist with such discussions. None of this information, however, diminishes medical physicists’ duty to ensure that CT exams are optimized for the patient's body habitus and the clinical indication.

## AUTHOR CONTRIBUTIONS

All authors have reviewed and approved the final document and provided editorial input and met all ICJME requirements for authorship.

## CONFLICT OF INTEREST STATEMENT

C.H.M. is the recipient of a research grant to the institution from Siemens Healthineers, unrelated to this work.
