# Correction to “CT scanning and questions about benefit versus risk”

**DOI:** 10.1002/acm2.70214

**Published:** 2025-08-26

McCollough CH, Milman R. CT scanning and questions about benefit versus risk. *J Appl Clin Med Phys*. 2025;26(7):e70179. doi:10.1002/acm2.70179

In the author byline, Dr Rebecca Milman's name was misspelled as Rebecca Millman. The online version of this article has been corrected accordingly.

We apologize for this error.

